# Patient Perspectives on the Usefulness of an Artificial Intelligence–Assisted Symptom Checker: Cross-Sectional Survey Study

**DOI:** 10.2196/14679

**Published:** 2020-01-30

**Authors:** Ashley N D Meyer, Traber D Giardina, Christiane Spitzmueller, Umber Shahid, Taylor M T Scott, Hardeep Singh

**Affiliations:** 1 Center for Innovations in Quality, Effectiveness and Safety Michael E DeBakey Veterans Affairs Medical Center and Baylor College of Medicine Houston, TX United States; 2 Department of Psychology, University of Houston Houston, TX United States

**Keywords:** clinical decision support systems, technology, diagnosis, patient safety, symptom checker, computer-assisted diagnosis

## Abstract

**Background:**

Patients are increasingly seeking Web-based symptom checkers to obtain diagnoses. However, little is known about the characteristics of the patients who use these resources, their rationale for use, and whether they find them accurate and useful.

**Objective:**

The study aimed to examine patients’ experiences using an artificial intelligence (AI)–assisted online symptom checker.

**Methods:**

An online survey was administered between March 2, 2018, through March 15, 2018, to US users of the Isabel Symptom Checker within 6 months of their use. User characteristics, experiences of symptom checker use, experiences discussing results with physicians, and prior personal history of experiencing a diagnostic error were collected.

**Results:**

A total of 329 usable responses was obtained. The mean respondent age was 48.0 (SD 16.7) years; most were women (230/304, 75.7%) and white (271/304, 89.1%). Patients most commonly used the symptom checker to better understand the causes of their symptoms (232/304, 76.3%), followed by for deciding whether to seek care (101/304, 33.2%) or where (eg, primary or urgent care: 63/304, 20.7%), obtaining medical advice without going to a doctor (48/304, 15.8%), and understanding their diagnoses better (39/304, 12.8%). Most patients reported receiving useful information for their health problems (274/304, 90.1%), with half reporting positive health effects (154/302, 51.0%). Most patients perceived it to be useful as a diagnostic tool (253/301, 84.1%), as a tool providing insights leading them closer to correct diagnoses (231/303, 76.2%), and reported they would use it again (278/304, 91.4%). Patients who discussed findings with their physicians (103/213, 48.4%) more often felt physicians were interested (42/103, 40.8%) than not interested in learning about the tool’s results (24/103, 23.3%) and more often felt physicians were open (62/103, 60.2%) than not open (21/103, 20.4%) to discussing the results. Compared with patients who had not previously experienced diagnostic errors (missed or delayed diagnoses: 123/304, 40.5%), patients who had previously experienced diagnostic errors (181/304, 59.5%) were more likely to use the symptom checker to determine where they should seek care (15/123, 12.2% vs 48/181, 26.5%; *P*=.002), but they less often felt that physicians were interested in discussing the tool’s results (20/34, 59% vs 22/69, 32%; *P*=.04).

**Conclusions:**

Despite ongoing concerns about symptom checker accuracy, a large patient-user group perceived an AI-assisted symptom checker as useful for diagnosis. Formal validation studies evaluating symptom checker accuracy and effectiveness in real-world practice could provide additional useful information about their benefit.

## Introduction

### Background

Patients are increasingly seeking to be more involved in their health care [1,2]. As a result, digital health care tools (both online and mobile health tools) have proliferated [3,4], and their use by patients has dramatically increased [5]. Overall, 1 in 3 US adults reported going online to try to self-diagnose a medical condition in 2013 [6]. In addition to searching the internet for health information, use of digital health care tools includes online, artificial intelligence (AI)–assisted symptom checkers for obtaining diagnoses or self-triage [7-10]. A previous report assessed the accuracy of general symptom checkers using patient vignettes [9] and found that diagnostic accuracy (defined as the correct diagnosis being listed first) was 34% and triage advice was appropriate 57% of the time. Accuracy varied considerably among symptom checkers (with a range of 5%-50%), leading to a concern about their use [11,12]. Furthermore, it is unknown if patients [7] use online symptom checkers as a replacement for seeing physicians in person. Also unknown are the rationale why patients use symptom checkers, whether they find them accurate and useful, and if these tools provide them with any benefit.

In light of evidence that approximately 1 in 20 US adults experience a diagnostic error every year (with half incurring severe or permanent harm) [13], the National Academies of Sciences, Engineering, and Medicine recommends the use of patient engagement tools, including symptom checkers and other digital health tools, in efforts to address this issue [14]. As a part of the solution digital health tools offer patients broader, quicker access to health information, [15], but their use may differ among patient groups. Mobile phone use for looking up general health information differs across race and ethnicity (with 67% of African Americans/blacks, 73% of Hispanics, and 58% of whites reportedly doing so) [16] and patients with chronic health conditions tend to have less access to the internet [17]. It is unclear how these patterns would relate to the use of online symptom checkers, but differences in use among these groups of patients could result in disparate benefits of the tools. Furthermore, other patient characteristics, such as previous positive or negative health care experiences, could also alter use, usefulness, and experiences with such tools.

Currently, it is unclear if patients use symptom checkers to supplement medical advice (which is what many of the tool developers suggest in addition to speaking with physicians about the obtained results) or if they are using them as a substitute for in-person health care by seeking in-person health care only if instructed by the symptom checker. Finally, in assessing symptom checker benefits, it is vital to understand patient perspectives [18] after actual use [19] (rather than to just assess their accuracy in fictitious situations as these data may not be ecologically valid). Knowledge about both the benefits of symptom checkers and how they can be improved could maximize patient benefits and minimize unintended consequences (such as *cyberchondria*, anxiety, or unnecessary health care use—proposed consequences of Web-based medical tools) [20-22].

### Objectives

To address current knowledge gaps, we examined user characteristics and experiences and potential consequences of symptom checker use, including subsequent physician discussions around use of the symptom checker in relation to a popular online AI-assisted symptom checker, the Isabel Symptom Checker [23]. In addition, we compared perceptions of the symptom checker in patients who previously experienced errors in diagnosis versus those who did not, because these experiences may affect symptom checker favorability.

## Methods

### Description of the Isabel Symptom Checker

**Figure 1:**
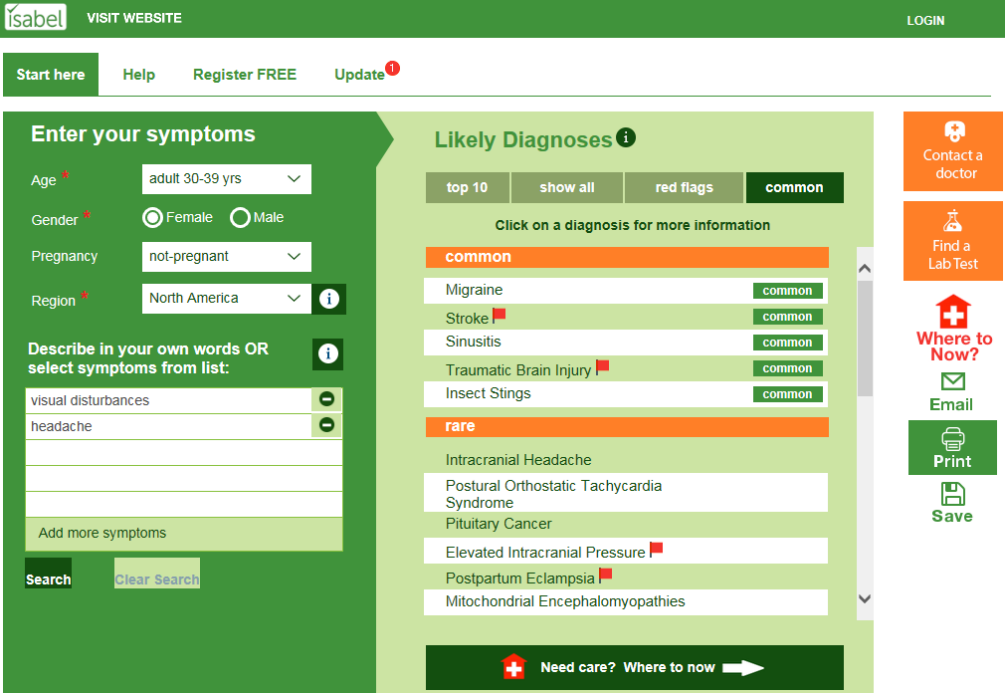
Screenshot of the patient facing, artificial intelligence assisted Isabel Symptom Checker.

The Isabel Symptom Checker (Isabel Healthcare) [23] is a free Web-based, AI-assisted symptom checker intended for use by patients (as opposed to the Isabel Differential Diagnosis Generator [Isabel Healthcare] intended for clinicians) and has been shown to have better accuracy than the average symptom checker in a vignette-based study (defined as having the correct diagnosis listed first in 44% of cases compared with an average rate of 34% in the 23 symptom checkers tested) [9]. It currently has over 12,000 registered users globally, with almost 7000 in the United States (not all users register) and the symptom checker completes between 200,000 to 300,000 searches per month [24]. Patients research their symptoms by entering their age range, gender, pregnancy status, geographic location or travel history, and symptoms in everyday language. Using machine learning and a training database of 6000 disease presentations, the symptom checker uses evidence-based natural language processing techniques to create a list of likely diagnoses ranked in order of relevance for the symptoms entered. Patients can sort their likely diagnoses as a top-10 list; a full list of all relevant diagnoses; a list including only red-flag, *do-not-miss* diagnoses, which indicate that medical advice should be sought immediately; or as a list divided into common versus rare diagnoses. Diagnoses are linked to reference resources, allowing patients to learn more. These resources include the consumer-facing Merck Manual (Merck Sharpe & Dohme Corp) [25], MedlinePlus (National Library of Medicine) [26], a patient version of UpToDate (UpToDate, Inc) [27], and the Mayo Clinic website (Mayo Foundation for Medical Education and Research) [28]. Next steps are provided where users can “*contact a doctor,”* “*find a lab test,”* or determine where they should go for medical care using additional triage functionality (using the “*Where Now”* button). The symptom checker is freely available and provides information for both adult and pediatric patients (see Figure 1 for example screenshot).

### Participants

With the help of Isabel Healthcare, we sent email invitations to all registered US users of the Isabel Symptom Checker (4000) to complete an online survey through SurveyMonkey (SurveyMonkey) [29], a commercial survey website. All of these users had registered and used the symptom checker within the last 6 months. On the basis of the limited available internal institutional funding, we were able to offer a survey incentive to only the first 385 respondents, all of whom received US $20 gift card incentives; these available funds thus determined sample size. Local institutional review board approval was obtained at Baylor College of Medicine and written consent was obtained from the participants.

### Survey

The survey was created by a multidisciplinary team (authors AM, TG, CS, and HS) with expertise in patient experience, cognitive psychology, psychometrics, internal medicine, and diagnostic errors. It comprises multiple-choice questions, 5-item Likert-type questions (with choices ranging from *strongly* *disagree* to *strongly agree*), and 5 open-ended questions and was designed to elicit information related to 4 main areas (see Multimedia Appendix 1 for full survey):

1. User characteristics (including age, gender, race, level of education, household income, and presence of chronic health conditions; see Multimedia Appendix 2 for full list).
2. Experiences of symptom checker use (including why and how patients used the tool, self-reported health and financial outcomes related to its use [in multiple-choice, Likert-type, and open-ended versions], whether they thought the symptom checker gave them useful information for their health problem, whether they followed the symptom checker’s advice to go to the emergency department (ED) if advised to do so, how easy to use and useful the tool was, and whether the tool led them closer to correct diagnoses; see Multimedia Appendix 3 for full list).
3. Experiences discussing symptom checker results with physicians (whether patients discussed symptom checker use with physicians, and if so, physicians’ receptiveness to patients’ use of the tool [including an open-ended question further detailing those experiences], and if not, why they chose not to [in both multiple-choice and open-ended versions]; see Multimedia Appendix 4 for full list).
4. Personal experience of an error in diagnosis previously (defined for them as whether or not they have ever been given either the wrong diagnosis for a health concern or not given any diagnosis for a health concern that they were seeking medical help for; this includes both multiple-choice questions and an open-ended response, where participants could detail their diagnostic error experiences).

After development, the survey was pilot tested in both paper and online forms with 5 and 13 patients, respectively, and correspondingly refined to increase readability and understandability by simplifying and clarifying the language.

### Data Analysis

All data were summarized using descriptive statistics, except open-ended responses, which were coded using content analysis. In addition, we compared demographics, experiences around Isabel Symptom Checker use, and subsequent interactions with physicians between users who had previously experienced diagnostic errors and those who had not using independent *t* tests, Chi-square, or Fisher exact tests where appropriate. We also conducted additional subanalyses using Chi-square or Fisher exact tests to determine whether certain behaviors (following the advice of the symptom checker and going to the ED and talking to one’s doctor about Isabel results) were associated with other demographics. All tests were two tailed, done using IBM SPSS Statistics 22 (IBM Corporation), and considered significant when *P*<.05.

## Results

### Sample

From the sample of 385 respondents, 329 provided mostly complete (>90% of the survey was complete) and relevant data (18 participants’ responses were excluded for not completing >90% of the questions and 38 because they described using the tool as a medical professional for either education or diagnosing patients when elaborating on the question “What prompted you to use the Isabel Symptom Checker?” after choosing the “Other” response). Only data from the 329 nonexcluded respondents are reported. The mean time to complete the survey was 12:21 (SD 10:43) min.

### User Characteristics

Mean respondent age was 48.0 (SD 16.7) years; most of them were women (230/304, 75.7%), white (271/304, 89.1%), with bachelor’s degrees or higher (191/302, 63.2%), and had less than US $100,000 in household income (216/287, 75.3%), health care coverage (296/304, 97.4%), and chronic health conditions (216/329, 65.7%; Multimedia Appendix 2).

### Experiences Around Symptom Checker Use

Patients most commonly used the symptom checker to better understand what could cause their symptoms (232/304, 76.3%). The next most common reasons included to decide whether to seek in-person health care (101/304, 33.2%), to decide what health care setting to visit (eg, primary or urgent care: 63/304, 20.7%), to get medical advice without going to the doctor (48/304, 15.8%), or to better understand the diagnosis made by their doctor (39/304, 12.8%). Many respondents used the symptom checker before (119/304, 39.1%) or both before *and* after seeing a physician (113/304, 37.2%). Of additional note, of the 26 patients given advice to proceed to the ED, 14 (54%) did. Most users thought the symptom checker gave them useful information for their health problems (274/304, 90.1% either strongly agreeing or agreeing) with about half reporting positive health effects (154/302, 51.0%). Although over half were neutral in terms of benefitting financially (172/303, 56.8%); most found the symptom checker useful: perceiving it to be satisfying (263/304, 86.5%), easy to use (182/303, 60.1%), useful as a diagnostic tool (253/301, 84.1%), and providing them with insights leading them closer to correct diagnoses (231/303, 76.2%). In addition, most reported they would use it again (278/304, 91.4%; Multimedia Appendix 3).

Open-ended responses detailing effects on health based on what participants learned most often included positive consequences (168/175, 96.0%). Most often, patients conveyed that symptom checker use enabled them to determine whether their condition might be serious, which helped them distinguish when to seek medical attention based on symptoms and severity (49/175, 28.0%; see Multimedia Appendix 5 for additional findings). Similarly, open-ended responses about financial effects were mostly positive (64/69, 93%) and most often related to reporting fewer doctor visits post–symptom checker use (34/69, 49%; see Multimedia Appendix 5 for additional findings).

### Experiences Discussing Symptom Checker Results With Physicians

Of those who visited physicians after using the tool (213/304, 70.1%), almost half discussed the findings with their physicians (103/213, 48.4%). Their experiences were mixed, but patients more often felt physicians were interested (42/103, 40.8%) than *not* interested (24/103, 23.3%) in learning about the tool’s results. Similarly, patients more often felt their physicians were open (62/103, 60.2%) than *not* open (21/103, 20.4%) to discussing the tool’s results (Multimedia Appendix 4). In open-ended responses, patients described both positive (15/29, 52%) and negative (14/29, 48%) interactions with their doctors when discussing their Isabel results. For example, the most often talked about positive experience discussing the results with physicians was the perception that physicians were open to the use of Isabel (6/29, 21%), yet the most often talked about negative experience was frustration on behalf of the patients during such discussions (7/29, 24%; see Multimedia Appendix 5 for additional findings).

Patients who chose *not* to discuss the findings with their physicians (110/213, 51.6%) did so because of various concerns, including thinking their doctors would not approve of their use of the tool or the doctors would think the patients mistrusted them or were trying to second guess or replace them by using the tool (see Multimedia Appendix 4). In the corresponding open-ended response, they most often described not discussing the results with their doctors because of worry about pushback or concerns about their physicians’ reactions (21/52, 40%; see Multimedia Appendix 5 for additional findings).

### Previous Experiences of Diagnostic Errors

More than half of the patients reported previously experiencing diagnostic errors (181/304, 59.5%). Females made up 80.7% (146/181) of the diagnostic error group but only 68.3% (84/123) of the nonerror group (see Multimedia Appendix 2; *P*=.01)*.* In addition, patient users reporting previous diagnostic errors reported having more doctor visits in the last year (10.4 vs 4.1 visits; *P*<.001); had higher use of online resources to obtain medical information, including sources other than WebMD or Google (35/181, 19.3% vs 10/123, 8.1%; *P*=.01); and were more likely to have arthritis (88/169, 52.1% vs 38/121, 31.4%; *P*<.001), asthma (47/166, 28.3% vs 15/117, 12.8%; *P*=.002), or other chronic health conditions (93/181, 51.4% vs 25/123, 20.3%; *P*<.001) compared with the nonerror group (Multimedia Appendix 2).

Users who previously experienced diagnostic errors were also more likely to use the Isabel Symptom Checker to determine where they should seek care (48/181, 26.5% vs 15/123, 12.2%; *P*=.002) and to use it both before and after seeing a doctor (rather than at only one time point; 86/181, 47.5% vs 27/123, 22.0%; *P*<.001). They were also more likely to experience positive health benefits from the symptom checker compared with others (98/181, 54.1% vs 56/121, 46.3%; *P*=.03). The diagnostic error group was also more likely to perceive they obtained insights about their diagnoses from the tool (141/180, 78.3% vs 90/123, 73.2%; *P*=.01), and less often found their doctors supportive regarding their use of the tool (61/179, 34.1% vs 48/121, 39.7%; *P*=.02; Multimedia Appendix 3).

Users who previously experienced diagnostic errors were more likely to see a doctor after using the symptom checker than those who did not (145/181, 80.1% vs 68/123, 55.3%; *P*<.001), but they were equally likely to discuss the results with their physicians (69/145, 47.6% vs 34/68, 50.0%; *P*=.74). In these conversations, however, they less often felt their doctors were interested in learning about their symptom checker results (22/69, 32% vs 20/34, 59%; *P*=.04; Multimedia Appendix 4).

When describing their diagnostic errors in open-ended responses (n=108), patients reported several contributory factors to their diagnostic errors. These included their perceptions that physicians (1) were unable to manage diagnostic uncertainty (33/108, 30.6%), (2) made multiple unnecessary referrals to others when faced with challenging diagnoses (22/108, 20.4%), (3) prioritized financial gains over patient benefit (19/108, 17.6%), (4) unfairly labelled patients (eg, as drug or attention seekers, as *drama queens*, as having symptoms “all in [their] head[s],” or as not “look[ing] sick”: 16/108, 14.8%), and (5) did not take the time to listen to patients (13/108, 12.0%). Several patients reported harm, including long-term health consequences from errors, such as disability or life-threatening experiences (73/108, 67.6%; see Multimedia Appendix 5 for additional findings).

### Additional Behavioral Differences as Related to Demographics

Neither likelihood of going to the ED when the symptom checker suggested (n=25) nor the likelihood of discussing the results with their doctors (assuming they saw a doctor after using the symptom checker; n=217) were significantly related to gender, income, education, or being an underrepresented minority in our sample (see Multimedia Appendix 5 for details).

## Discussion

### Principal Findings

Patients used an online symptom checker to learn more about what could cause their symptoms, to determine whether to seek care or where, to get medical advice without going to a doctor, or to better understand their diagnosis. Most patients thought the tool gave them useful information for their health problems and thought it provided them with insights leading them closer to correct diagnoses. Half of the patients reported positive health effects. However, the patients who discussed the findings with their physicians conveyed mixed experiences about whether physicians were interested or open about discussing symptom checker results.

### Strengths

The strengths of this study are the examination of naturalistic patient experiences and the assessment of subsequent related events, which are often missing from existing digital health tool studies (most previous studies examined vignette-based assessments [30,31] or patients already presenting to their doctors [32-35] with limited follow-up) [7]. Most patients used the symptom checker between 2 weeks and 4 months before the survey, allowing for adequate time for diagnoses to evolve and related subsequent events to occur, such as the completion of diagnostic tests, referrals, treatment, and potential responses to treatment.

### Limitations

However, there are several study limitations. As we rely on self-reported data, there is no validation of patient outcomes via some type of medical record audit, making it difficult to assess outcome accuracy. Nonetheless, over time, patients would have enough information to make a determination about the ultimate accuracy of the diagnosis suggested by the tool. In addition, as with all surveys, participants may be subject to acquiescence bias—the tendency to agree with most statements. However, we did not find much evidence for this: despite much agreement with positively worded questions, negatively worded questions were not similarly agreed with (people were not merely agreeing). An additional limitation is that these data represent patient perceptions of only 1 symptom checker, and it is not clear if these results would generalize to other symptom checkers, especially to those that do not utilize AI-assisted natural language searching. We also offered an incentive of a US $20 gift card to the first 385 participants, which may have skewed our sample to people who are quick to respond to emails. Our sample might also be unique: participants had a mean of 8 visits to physicians within the last 12 months, meaning they could be different—perhaps sicker—compared with the general population. However, this population may also be more likely to use such tools given their high interaction with the health care system, so these patterns are still important to understand. In addition, our sample is overwhelmingly female and white, with a mean age of 48 years, thereby reducing our ability to examine demographic differences in terms of experiences or behavior related to symptom checker use. However, this represents user data available from Isabel Healthcare (females represented 62% of users over the last year, with 39% of users aged between 40 and 64 years). It is difficult to know if our sample is representative of typical users in other ways. Finally, this study was not designed to explain the differences in perceptions and experiences between groups who had experienced diagnostic errors versus those who had not, but only to describe them: the reasons for these differences are likely very complicated and future studies could further examine the roots of these differences.

### For Additional Discussion

Some findings warrant additional consideration. For example, previous studies show that some underrepresented groups use mobile resources more for obtaining health information [16]. Perhaps these groups are using digital health tools as a substitute for other less-available health resources. Given that the long-term implications of using these tools are not understood, this could represent disparities affecting health outcomes, especially as patients in this study used the tool to triage themselves or get medical advice without going to a doctor. Nonetheless, our sample did not overwhelmingly include underrepresented groups. As such, additional research is needed to further scrutinize disparities related to symptom checker use.

Another finding worth additional consideration is that over half of the respondents reported previously experiencing diagnostic errors. Although this may seem high, this is a selected sample of symptom checker users, many of whom have had multiple interactions with the health care system. We do not intend this to be a population-based estimate. Nonetheless, the National Academies of Sciences, Engineering, and Medicine have extrapolated from large estimates that most Americans will get a wrong or late diagnosis at some point in their lives [14], and population-based surveys suggest that 12% of patients may have been misdiagnosed, so the high rate of misdiagnosis is quite possible in our sample [36]. These patients used the tool at more time points and used more online health resources in general, but they perceived their doctors to be less interested when discussing the tool’s results. This could relate to the higher incidence of chronic diseases reported in this group and more negative health care experiences that often occur in patients with chronic disease [37]. Although past dissatisfaction with the health care system has been linked to increased use of the internet for health-related purposes [38-40], the impact of medical circumstances or past diagnostic errors on the use of alternate health resources (such as symptom checkers) remains ripe for exploration.

Our findings also highlight a disconnect between patients and physicians when it comes to the use of digital health tools. Although the sample was generally enthusiastic and satisfied with the tool, the patients felt their physicians showed mixed receptivity to the information and mixed openness to discussing it. This might discourage future use of such tools and future engagement by patients, similar to patterns seen in the contrasting patient and physician enthusiasm about email use for health communications [41].

In addition to this concern, a fear that has surfaced over the use of these tools is the potential for patients’ anxiety to increase, thereby increasing health care utilization. These data show that many patients are using the tool to see whether they needed to see a doctor and help them determine where they should seek care. Despite this, a previous study pointed out that this particular symptom checker never advises self-care, which may also increase health care utilization [9]. We currently do not know if such tools would lead to a significant increase in health care use. A larger sample and additional objective follow-up data would help us understand if this represents appropriate utilization of resources.

Finally, we think it is worth reflecting on the effect that such tools might have on patients’ sense of confidence in their abilities to diagnose themselves. Diagnosis is a task that often involves clinical uncertainty, something physicians themselves face [42]. Undoubtedly, patients would experience more diagnostic uncertainty than physicians owing to less expertise, but as more patients use these types of tools and obtain answers without actually seeing a health care professional, it will be important to examine the effect of these tools on how patients think about self-diagnosis and any resulting consequences thereof (such as false reassurance, suggested by others [43]). This study is an initial examination of real-life symptom checker use, but as Fraser et al point out [43], the evaluation of such tools should assess them with increasing ecological validity and should examine multiple aspects: usability, effectiveness, and safety. We have begun to examine usability and effectiveness, but much more remains to be understood to thoroughly investigate all of these facets in real-world situations.

### Conclusions

In conclusion, while accessing a popular online symptom checker for triage and diagnosis, patients reported receiving useful information for their diagnostic process, despite ongoing concerns about the accuracy of various types of symptom checkers [43]. Prior negative health care experiences related to misdiagnoses might affect how patients use and benefit from these tools for triage and diagnosis, an area ripe for exploration. Evaluation of long-term, objective health benefits, particularly in diverse patient groups, is needed to better understand the broader impact of symptom checkers on diagnosis and health outcomes.

## Appendix

Multimedia Appendix 1 Full survey (delivered via SurveyMonkey): Isabel Symptom Checker Post-Use Survey.

Multimedia Appendix 2 Characteristics of the Isabel Symptom Checker patient users.

Multimedia Appendix 3 Patient experiences of using the Isabel Symptom Checker.

Multimedia Appendix 4 Patients’ experiences of discussing the Isabel Symptom Checker results with their physicians.

Multimedia Appendix 5 Additional findings from open-ended responses.

## Abbreviations

AI: artificial intelligence
ED: emergency department
HSR&D: Health Services Research and Development
VA: Department of Veterans Affairs

## References

[1] McDonald KM, Bryce CL, Graber ML (2013). The patient is in: patient involvement strategies for diagnostic error mitigation. BMJ Qual Saf.

[2] Anderson M, McCleary KK (2015). From passengers to co-pilots: patient roles expand. Sci Transl Med.

[3] Ralf-Gordon J (2016). Research 2 Guidance.

[4] Aitken M, Lyle J (2015). IQVIA.

[5] Powell AC, Landman AB, Bates DW (2014). In search of a few good apps. J Am Med Assoc.

[6] Fox S, Duggan M (2013). Pew Research Center.

[7] Millenson ML, Baldwin JL, Zipperer L, Singh H (2018). Beyond Dr. Google: the evidence on consumer-facing digital tools for diagnosis. Diagnosis (Berl).

[8] Jutel A, Lupton D (2015). Digitizing diagnosis: a review of mobile applications in the diagnostic process. Diagnosis (Berl).

[9] Semigran HL, Linder JA, Gidengil C, Mehrotra A (2015). Evaluation of symptom checkers for self diagnosis and triage: audit study. Br Med J.

[10] Wyatt JC (2015). Fifty million people use computerised self triage. Br Med J.

[11] Kao CK, Liebovitz DM (2017). Consumer mobile health apps: current state, barriers, and future directions. Phys Med Rehabil.

[12] Bauer M, Glenn T, Monteith S, Bauer R, Whybrow PC, Geddes J (2017). Ethical perspectives on recommending digital technology for patients with mental illness. Int J Bipolar Disord.

[13] Singh H, Meyer AN, Thomas EJ (2014). The frequency of diagnostic errors in outpatient care: estimations from three large observational studies involving US adult populations. BMJ Qual Saf.

[14] National Academies of Sciences, Engineering, and Medicine, Institute of Medicine, Board on Health Care Services, Committee on Diagnostic Error in Health Care (2015). Improving Diagnosis in Health Care.

[15] Akter S, Ray P (2010). mHealth - an ultimate platform to serve the unserved. Yearb Med Inform.

[16] Anderson M (2015). Pew Research Center.

[17] Fox S, Duggan M (2013). Pew Research Center.

[18] Vincent CA, Coulter A (2002). Patient safety: what about the patient?. Qual Saf Health Care.

[19] McCartney M (2017). Margaret McCartney: innovation without sufficient evidence is a disservice to all. Br Med J.

[20] White RW, Horvitz E (2009). Cyberchondria: studies of the escalation of medical concerns in web search. ACM Trans Inf Syst.

[21] Mueller J, Jay C, Harper S, Davies A, Vega J, Todd C (2017). Web use for symptom appraisal of physical health conditions: a systematic review. J Med Internet Res.

[22] Gass MA (2016). Digital Commons at Salem State University.

[23] (2018). Isabel Symptom Checker.

[24] Maude J (2018). Meyer AN.

[25] (2019). Merck Manuals.

[26] (2019). MedlinePlus.

[27] (2019). UpToDate.

[28] Mayo Foundation for Medical Education Research (MFMER) (2019). Mayo Clinic.

[29] (2019). SurveyMonkey.

[30] Coiera E (2018). The Guide to Health Informatics 3rd Edition.

[31] Luger TM, Houston TK, Suls J (2014). Older adult experience of online diagnosis: results from a scenario-based think-aloud protocol. J Med Internet Res.

[32] Powley L, McIlroy G, Simons G, Raza K (2016). Are online symptoms checkers useful for patients with inflammatory arthritis?. BMC Musculoskelet Disord.

[33] Bisson LJ, Komm JT, Bernas GA, Fineberg MS, Marzo JM, Rauh MA, Smolinski RJ, Wind WM (2016). How accurate are patients at diagnosing the cause of their knee pain with the help of a web-based symptom checker?. Orthop J Sports Med.

[34] Hageman MG, Anderson J, Blok R, Bossen JK, Ring D (2015). Internet self-diagnosis in hand surgery. Hand (N Y).

[35] Farmer SE, Bernardotto M, Singh V (2011). How good is internet self-diagnosis of ENT symptoms using Boots WebMD symptom checker?. Clin Otolaryngol.

[36] NORC at the University of Chicago, IHI/NPSF Lucian Leape Institute (2017). Americans' Experiences with Medical Errors and Views on Patient Safety. Institute for Healthcare Improvement.

[37] Ali A, Vitulano L, Lee R, Weiss TR, Colson ER (2014). Experiences of patients identifying with chronic Lyme disease in the healthcare system: a qualitative study. BMC Fam Pract.

[38] de Rosis S, Barsanti S (2016). Patient satisfaction, e-health and the evolution of the patient-general practitioner relationship: evidence from an Italian survey. Health Policy.

[39] Li N, Orrange S, Kravitz RL, Bell RA (2014). Reasons for and predictors of patients' online health information seeking following a medical appointment. Fam Pract.

[40] Tustin N (2010). The role of patient satisfaction in online health information seeking. J Health Commun.

[41] Singh H, Fox SA, Petersen NJ, Shethia A, Street RL (2009). Older patients' enthusiasm to use electronic mail to communicate with their physicians: cross-sectional survey. J Med Internet Res.

[42] Bhise V, Rajan SS, Sittig DF, Morgan RO, Chaudhary P, Singh H (2018). Defining and measuring diagnostic uncertainty in medicine: a systematic review. J Gen Intern Med.

[43] Fraser H, Coiera E, Wong D (2018). Safety of patient-facing digital symptom checkers. Lancet.

