# Supplementary material for: Patient Perspectives on the Usefulness of an Artificial Intelligence–Assisted Symptom Checker: Cross-Sectional Survey Study
Source: J Med Internet Res. 2020 Jan 30;22(1):e14679. doi: 10.2196/14679 (PMC7055765; doi:10.2196/14679)
Supplement: Multimedia Appendix 1 [file jmir_v22i1e14679_app1.docx]

**Multimedia Appendix 1 for Patient Perspectives on Usefulness of an Artificial-Intelligence Assisted Symptom Checker: Cross Sectional Survey Study**

Ashley N.D. Meyer^1^; Traber D. Giardina^1;^ Christiane Spitzmueller^2^; Umber Shahid, DrPH^1^; Taylor M.T. Scott, BA^1^; & Hardeep Singh^1^

^1^Center for Innovations in Quality, Effectiveness and Safety, Michael E. DeBakey Veterans Affairs Medical Center and Baylor College of Medicine, Houston, TX

^2^Department of Psychology, University of Houston, Houston, TX

**Full Survey (Delivered via SurveyMonkey)**

**Isabel Symptom Checker Post-Use Survey**

**1. Survey Background**

Welcome to our survey about the Isabel Symptom Checker! Isabel is collaborating with us at Baylor College of Medicine in an effort to find out more about your experiences using the Isabel Symptom Checker, an online medical diagnosis and assessment tool for patients. Your participation in this survey will serve as an important tool for improving online diagnostic tools to where they can better serve patients. No foreseeable risks or discomforts are associated with participating in this study. There are no immediate benefits to you for participating in this study, but you will receive $20 in Barnes & Noble gift cards for completing the survey.

Completing this survey will take between 10 and 15 minutes. Please remember that participation in

this survey is entirely voluntary and non-participation will not result in penalty or loss of benefits to

which you would otherwise be entitled.

The survey asks a number of questions regarding your opinions and experiences with the Isabel

Symptom Checker.

Ashley Meyer, PhD (a cognitive psychologist) and Hardeep Singh, MD (an internal medicine

physician and patient safety researcher) serve as the principal investigators of this project. All data

will be treated confidentially. Only aggregate results at the group level will be reported back to

Isabel or published. No individual-level data will be reported or given to anyone at Isabel or any

other entity. The data from this study will be stored securely at Baylor College of Medicine.

Only members of the PIs' team will have access to this data. Data from this survey may be included

in journal publications in the domain of health services research.

This project has been reviewed by Baylor College of Medicine's Institutional Review Board for the

Protection of Human Subjects (713)798-6970. If you have questions or trouble accessing the

website and would like assistance, please contact Dr. Ashley Meyer by email at ameyer@bcm.edu.

Your honest answers are important to this project and to the future of online diagnostic tools. We

thank you in advance for your participation!

Isabel Symptom Checker

1) If you agree to participate in the current study, please insert your first and last name initials below as confirmation of your consent to participate.

**_________________________**

**The following questions pertain to your experiences with Isabel Symptom Checker.**

2) Approximately how long ago did you use Isabel Symptom Checker?

1. 0-2 weeks ago
2. 2-4 weeks ago
3. 1-2 months ago
4. 3-4 months ago
5. More than 4 months ago
6. What prompted you to use Isabel Symptom Checker? I wanted to ____(Choose all that apply)
   - see whether or not I needed to see a doctor
   - better understand what could be causing my symptoms
   - get medical advice without going to the doctor
   - better understand the diagnosis made by my doctor
   - help me determine where I should seek care
   - other (please specify) ____________________________________________________________
7. When did you use Isabel Symptom Checker?
   1. before seeing a doctor
   2. after seeing a doctor
   3. both before and after seeing a doctor
   4. instead of seeing a doctor
8. When I used Isabel Symptom Checker, I had health insurance or health care coverage (e.g., employer based, VA care, Medicare or Medicaid).
   1. Yes
   2. No
9. **Please indicate the extent to which you agree or disagree with the following statements regarding your experiences with Isabel Symptom Checker.**

|  | Strongly disagree | Disagree | Neither agree nor disagree | Agree | Strongly agree |
| --- | --- | --- | --- | --- | --- |
| Before I used Isabel Symptom Checker, I was already planning on going to the doctor. | ○ | ○ | ○ | ○ | ○ |
| Personal financial issues prevented me from seeing a doctor in person for my health issues. | ○ | ○ | ○ | ○ | ○ |
| Policies related to my health insurance prevented me from seeing a doctor in person for my health issues. | ○ | ○ | ○ | ○ | ○ |
| I think Isabel Symptom Checker gave me useful information for my health problem. | ○ | ○ | ○ | ○ | ○ |
| I experienced positive effects on my health as a result of what I learned from using Isabel Symptom Checker. | ○ | ○ | ○ | ○ | ○ |
| I experienced positive effects on my finances as a result of what I learned from using Isabel Symptom Checker. | ○ | ○ | ○ | ○ | ○ |

1. Please describe any positive or negative effects on your health you experienced based on what you learned from using Isabel Symptom Checker.

[Text box]________________________________________________________________________

1. Please describe any positive or negative effects on your finances you experienced based on what you learned from using Isabel Symptom Checker.

[Text box]________________________________________________________________________

1. When given a list of possible diagnoses for my health problem, I read the relevant information provided by Isabel Symptom Checker about those diagnoses.
   1. Yes
   2. No
2. After seeing the list of possible diagnoses for my health problem, I used the ‘Where Now‘ feature to help me decide whether to see my doctor or go to the Emergency Room.
   1. Yes
   2. No
3. If the Isabel Symptom Checker suggested that you go to the Emergency Room (ER), did you follow that advice?
   1. Yes- Isabel Symptom Checker suggested I go to the ER and I went.
   2. No- Isabel Symptom Checker suggested I go to the ER, but I did not go to any medical care provider.
   3. No - Isabel Symptom Checker suggested I go to the ER, but I decided I didn’t need to go to the ER, but I did go to another medical care provider.
   4. Not Applicable- Isabel Symptom Checker did not suggest I go to the ER.
4. **Please rate your overall health.**

|  | 1- Not in good health | 2 | 3 | 4 | 5 | 6 | 7 | 8 | 9 | 10- In very good health |
| --- | --- | --- | --- | --- | --- | --- | --- | --- | --- | --- |
| Before using Isabel Symptom Checker | ○ | ○ | ○ | ○ | ○ | ○ | ○ | ○ | ○ | ○ |
| After using Isabel Symptom Checker | ○ | ○ | ○ | ○ | ○ | ○ | ○ | ○ | ○ | ○ |

1. **Please give us your opinion of the results Isabel Symptom Checker gave you by clicking on a response below.**

|  | Strongly disagree | Disagree | Neither agree nor disagree | Agree | Strongly agree |
| --- | --- | --- | --- | --- | --- |
| Some of the diagnoses the Isabel Symptom Checker suggested seemed more alarming than I thought they should have been given my symptoms. | ○ | ○ | ○ | ○ | ○ |
| Some of the diagnoses the Isabel Symptom Checker suggested seemed less alarming than I thought they should have been given my symptoms. | ○ | ○ | ○ | ○ | ○ |
| I am satisfied with Isabel Symptom Checker as an online diagnostic tool. | ○ | ○ | ○ | ○ | ○ |
| I think Isabel Symptom Checker was easy to use. | ○ | ○ | ○ | ○ | ○ |
| I found Isabel Symptom Checker very useful as a diagnostic tool. | ○ | ○ | ○ | ○ | ○ |
| The Isabel Symptom Checker provided me with insights that lead me closer to a correct diagnosis. | ○ | ○ | ○ | ○ | ○ |
| If I have a medical problem in the future, I would use Isabel Symptom Checker again. | ○ | ○ | ○ | ○ | ○ |
| I would recommend Isabel Symptom Checker to a friend or family member who wanted to research likely causes for their symptoms. | ○ | ○ | ○ | ○ | ○ |
| If asked, my doctor would support my decision to use Isabel Symptom Checker. | ○ | ○ | ○ | ○ | ○ |

1. AFTER you used Isabel Symptom Checker, did you see a doctor face-to-face to address the medical issue you were experiencing when you consulted Isabel Symptom Checker?
   1. Yes
   2. No
2. Did you discuss your Isabel Symptom Checker results with your doctor?
   1. Yes
   2. No
3. **Please indicate the extent to which you agree or disagree with the following statements regarding your experiences with Isabel Symptom Checker.**

|  | Strongly disagree | Disagree | Neither agree nor disagree | Agree | Strongly agree |
| --- | --- | --- | --- | --- | --- |
| My doctor was interested in learning about my results from the Isabel Symptom Checker. | ○ | ○ | ○ | ○ | ○ |
| My doctor was dismissive of Isabel Symptom Checker and similar tools. | ○ | ○ | ○ | ○ | ○ |
| My use of the Isabel Symptom Checker provided my doctor with relevant and helpful information. | ○ | ○ | ○ | ○ | ○ |
| My doctor seemed open to discussing my results from the Isabel Symptom Checker. | ○ | ○ | ○ | ○ | ○ |
| Based on my interactions with my doctor, I feel encouraged to use the Isabel Symptom Checker again in the future. | ○ | ○ | ○ | ○ | ○ |

1. Did you bring a printout of your Isabel Symptom Checker results to your doctor's appointment?
   1. Yes
   2. No
2. Are there additional things you would like us to know regarding your interaction with your doctor when discussing your results from the Isabel Symptom Checker? If yes, please describe below.

[Text box]________________________________________________________________________

19) Please indicate why you did not discuss your Isabel Symptom Checker results with your doctor.

|  | Strongly disagree | Disagree | Neither agree nor disagree | Agree | Strongly agree |
| --- | --- | --- | --- | --- | --- |
| I did not think my doctor would approve of my decision to use the Isabel Symptom Checker. | ○ | ○ | ○ | ○ | ○ |
| I was worried my doctor would feel I did not trust him/her if I brought information about the  Isabel Symptom Checker results to my appointment. | ○ | ○ | ○ | ○ | ○ |
| I did not want my doctor to feel I was trying to second guess or replace him/her by using the Isabel Symptom Checker. | ○ | ○ | ○ | ○ | ○ |

20) Are there additional things you would like us to know regarding your interaction with your doctor when discussing your results from the Isabel Symptom Checker? If yes, please describe below.

[Text box]________________________________________________________________________

21) Is there anything else you would like to share or comment about regarding your experiences with the Isabel Symptom Checker? If yes, please write them below.

[Text box]________________________________________________________________________

**General Questions About You**

This will give us an idea about who will benefit most from using the Isabel Symptom Checker and other online diagnostic tools. This information will be used for research purposes only.

22) What gender do you most closely identify with?

a) Female

b) Male

23) What is your age?

[Text box]________________________________________________________________________

24) What is your ethnicity? (Choose all that apply)

a) American Indian or Alaskan Native

b) Asian or Pacific Islander

c) Black or African American

d) Hispanic or Latino

e) White / Caucasian

f) Prefer not to answer

g) Other (please specify)

[Text box]______________________________________________________________________

25) What is the highest level of school you have COMPLETED?

a) Less than a high school diploma

b) High school graduate, GED, or alternative

c) Some college or associate’s degree

d) Bachelor’s degree

e) Graduate degree

26) What is your approximate annual household income?

a) $0-$24,999

b) $25,000-$49,999

c) $50,000-$74,999

d) $75,000-$99,999

e) $100,000-$124,999

f) $125,000-$149,999

g) $150,000-$174,999

h) $175,000-$199,999

i) $200,000 and up

27) In the past 12 months, how many times did you visit a doctor?

[Text box]________________________________________________________________________

28) How many miles away is your nearest hospital?

[Text box]________________________________________________________________________

29) Do you have any of the following chronic health conditions?

a) Hypertension

b) Coronary heart disease

c) Stroke

d) Diabetes

e) Cancer

f) Arthritis

g) Hepatitis

h) Weak or falling kidneys

i) Asthma

j) Chronic Obstructive Pulmonary Disease (COPD)

k) Other (please specify)

[Text box]________________________________________________________________________

30) Please indicate if you have used any of the listed online diagnostic tools to obtain medical information.

a) WebMD

b) Google

c) Other (please specify)

[Text box]________________________________________________________________________

31) Have you ever been misdiagnosed (either given the wrong diagnosis for a health concern or not given any diagnosis for a health concern that you were seeking medical help for)?

a) Yes

b) No

32) If you have been misdiagnosed and would like to share your story, please do so in the following textbox. This might help us figure out ways to help patients like you in the future. All of the information will be kept confidential.

[Text box]________________________________________________________________________

Thank you for participating in this survey about the Isabel Symptom Checker. Your answers will help inform future tools or improvements upon existing tools that help patients obtain diagnoses.

33) If you would like to receive a $20 Barnes & Noble gift card for completing the survey, please enter your email address here (your email address will not be tied to any of the data that was collected above, but will be needed to deliver the gift cards). Please allow 1-2 business days for gift card delivery.

[Text box]________________________________________________________________________
