# Supplementary material for: Patient Perspectives on the Usefulness of an Artificial Intelligence–Assisted Symptom Checker: Cross-Sectional Survey Study
Source: J Med Internet Res. 2020 Jan 30;22(1):e14679. doi: 10.2196/14679 (PMC7055765; doi:10.2196/14679)
Supplement: Multimedia Appendix 2 [file jmir_v22i1e14679_app2.docx]

**Multimedia Appendix 2 for Patient Perspectives on Usefulness of an Artificial-Intelligence Assisted Symptom Checker: Cross Sectional Survey Study**

Ashley N.D. Meyer^1^; Traber D. Giardina^1;^ Christiane Spitzmueller^2^; Umber Shahid, DrPH^1^; Taylor M.T. Scott, BA^1^; & Hardeep Singh^1^

^1^Center for Innovations in Quality, Effectiveness and Safety, Michael E. DeBakey Veterans Affairs Medical Center and Baylor College of Medicine, Houston, TX

^2^Department of Psychology, University of Houston, Houston, TX

**Characteristics of the Isabel Symptom Checker patient users**

| Characteristics^a^ | | Values | No reported diagnostic error | Reported diagnostic error | *P* value |
| --- | --- | --- | --- | --- | --- |
| Age (years; n=295), mean (SD) | | 48.0 (16.7) | 48.1 (17.8) (n=118) | 47.9 (16.0) (n=177) | .92 (*t* test) |
| **Gender (n=304), n (%)** | | | | | .01 (*χ^2^*) |
|  | Male | 74 (24.3) | 39 (31.7) | 35 (19.3) | — |
|  | Female | 230 (75.7) | 84 (68.3) | 146 (80.7) | — |
| **Race (could choose multiple options; n=304), n (%)** | | | | | |
|  | American Indian or Alaskan Native | 5 (1.6) | 0 (0.0) | 5 (2.8) | .08 (Fisher) |
|  | Asian or Pacific Islander | 13 (4.3) | 7 (5.7) | 6 (3.3) | .32 (*χ^2^*) |
|  | Black or African American | 7 (2.3) | 4 (3.3) | 3 (1.7) | .45 (Fisher) |
|  | Hispanic or Latino | 10 (3.3) | 4 (3.3) | 6 (3.3) | Approximately 1 (Fisher) |
|  | White/Caucasian | 271 (89.1) | 107 (87.0) | 164 (90.6) | .3 (*χ^2^*) |
|  | Other or unknown | 6 (2.0) | 3 (2.4) | 3 (1.7) | .69 (Fisher) |
| **Highest degree or level of education (n=302), n (%)** | | | | | .10 (*χ^2^*) |
|  | Less than high school diploma | 8 (2.6) | 5 (4.1) | 3 (1.7) | — |
|  | High school graduate or equivalent (e.g. General Educational Development test completion) | 14 (4.6) | 5 (4.1) | 9 (5.0) | — |
|  | Some college or associate’s degree | 89 (29.5) | 27 (22.0) | 62 (34.6) | — |
|  | Bachelor’s degree | 87 (28.8) | 42 (34.1) | 45 (25.1) | — |
|  | Graduate degree | 104 (34.4) | 44 (35.8) | 60 (33.5) | — |
| **Annual household income (n=287), n (%)** | | | | | .11 (*χ^2^*) |
|  | $0-$24,999 | 51 (17.8) | 17 (14.3) | 34 (20.2) | — |
|  | $25,000-$49,999 | 57 (19.9) | 19 (16.0) | 38 (22.6) | — |
|  | $50,000-$74,999 | 64 (22.3) | 34 (28.6) | 30 (17.9) | — |
|  | $75,000-$99,999 | 44 (15.3) | 19 (16.0) | 25 (14.9) | — |
|  | $100,000-$124,999 | 24 (8.4) | 9 (7.6) | 15 (8.9) | — |
|  | $125,000-$149,999 | 15 (5.2) | 8 (6.7) | 7 (4.2) | — |
|  | $150,000-$174,999 | 11 (3.8) | 2 (1.7) | 9 (5.4) | — |
|  | $175,000-$199,000 | 2 (0.7) | 2 (1.7) | 0 (0.0) | — |
|  | >$200,000 | 19 (6.6) | 9 (7.6) | 10 (6.0) | — |
| **Had health insurance or health care coverage (eg, employer based, Veterans Affairs care, Medicare, or Medicaid) when using Isabel Symptom Checker (n=304), n (%)** | | | | | .15 (Fisher) |
|  | No | 8 (2.6) | 1 (0.8) | 7 (3.9) | — |
|  | Yes | 296 (97.4) | 122 (99.2) | 174 (96.1) | — |
| Approximate number of visits to a doctor in last 12 months (n=301), mean (SD) | | 7.9 (9.0) | 4.1 (4.1) | 10.4 (10.4) | <.001 (*t* test, equal variances not assumed) |
| Approximate number of miles from nearest hospital (n=299), mean (SD) | | 7.5 (7.9) | 7.0 (6.7) | 7.8 (8.6) | .36 (*t* test equal variances not assumed) |
| **Has used any of the listed online diagnostic tools to obtain medical information (n=304), n (%)** | | | | | |
|  | WebMD | 239 (78.6) | 94 (76.4) | 145 (80.1) | .44 (*χ^2^*) |
|  | Google | 209 (68.8) | 86 (69.9) | 123 (68.0) | .72 (*χ^2^*) |
|  | Other | 45 (14.8) | 10 (8.1) | 35 (19.3) | .01 (*χ^2^*) |
| **Presence of chronic health conditions, n (%)** | | | | | |
|  | Hypertension (n=290) | 90 (31.0) | 31 (25.6) | 59 (34.9) | .09 (*χ^2^*) |
|  | Congestive heart failure (n=283) | 9 (3.2) | 3 (2.5) | 6 (3.7) | .74 (Fisher) |
|  | Stroke (n=283) | 10 (3.5) | 4 (3.4) | 6 (3.6) | ~1 (Fisher) |
|  | Diabetes (n=286) | 35 (12.2) | 10 (8.5) | 25 (14.9) | .10 (*χ^2^*) |
|  | Cancer (n=284) | 12 (4.2) | 2 (1.7) | 10 (6.0) | .13 (Fisher) |
|  | Arthritis (n=290) | 126 (43.4) | 38 (31.4) | 88 (52.1) | <.001 (*χ^2^*) |
|  | Hepatitis (n=282) | 2 (0.7) | 2 (1.7) | 0 (0.0) | .17 (Fisher) |
|  | Weak or failing kidneys (n=283) | 8 (2.8) | 2 (1.7) | 6 (3.6) | .48 (Fisher) |
|  | Asthma (n=283) | 62 (21.9) | 15 (12.8) | 47 (28.3) | .002 (*χ^2^*) |
|  | Chronic obstructive pulmonary disease (n=281) | 15 (5.3) | 4 (3.%) | 11 (6.6) | .26 (*χ^2^*) |
|  | Other chronic diseases (n=304) | 118 (38.8) | 25 (20.3) | 93 (51.4) | <.001 (*χ^2^*) |

^a^Not everyone answered every question, so sample sizes vary by patient characteristic. In examining characteristics by the presence or absence of reported diagnostic error, only patients who answered both questions are included.
