# Supplementary material for: Patient Perspectives on the Usefulness of an Artificial Intelligence–Assisted Symptom Checker: Cross-Sectional Survey Study
Source: J Med Internet Res. 2020 Jan 30;22(1):e14679. doi: 10.2196/14679 (PMC7055765; doi:10.2196/14679)
Supplement: Multimedia Appendix 3 [file jmir_v22i1e14679_app3.docx]

**Multimedia Appendix 3 for Patient Perspectives on Usefulness of an Artificial-Intelligence Assisted Symptom Checker: Cross Sectional Survey Study**

Ashley N.D. Meyer^1^; Traber D. Giardina^1;^ Christiane Spitzmueller^2^; Umber Shahid, DrPH^1^; Taylor M.T. Scott, BA^1^; & Hardeep Singh^1^

^1^Center for Innovations in Quality, Effectiveness and Safety, Michael E. DeBakey Veterans Affairs Medical Center and Baylor College of Medicine, Houston, TX

^2^Department of Psychology, University of Houston, Houston, TX

**Patient experiences of using the Isabel Symptom Checker**

| Variable^a^ | | Values | No reported diagnostic error | Reported diagnostic error | *P* value |
| --- | --- | --- | --- | --- | --- |
| **Approximately how long ago did you last use the Isabel Symptom Checker? (n=304), n (%)** | | | | | .98 (*χ^2^*) |
|  | 0-2 weeks ago^b^ | 79 (26.0) | 31 (25.2) | 48 (26.5) | — |
|  | 2-4 weeks ago^b^ | 72 (23.7) | 29 (23.6) | 43 (23.8) | — |
|  | 1-2 months ago | 74 (24.3) | 31 (25.2) | 43 (23.8) | — |
|  | 3-4 months ago | 39 (12.8) | 17 (13.8) | 22 (12.2) | — |
|  | More than 4 months ago | 40 (13.2) | 15 (12.2) | 25 (13.8) | — |
| **What prompted you to use the Isabel Symptom Checker? (could choose multiple options; n=304), n (%)** | | | | | |
|  | Better understand what could be causing my symptoms | 232 (76.3) | 92 (74.8) | 140 (77.3) | .61 (*χ^2^*) |
|  | See whether or not I needed to see a doctor | 101 (33.2) | 38 (30.9) | 63 (34.8) | .48 (*χ^2^*) |
|  | Help me determine where I should seek care | 63 (20.7) | 15 (12.2) | 48 (26.5) | .002 (*χ^2^*) |
|  | Get medical advice without going to the doctor | 48 (15.8) | 21 (17.1) | 27 (14.9) | .61 (*χ^2^*) |
|  | Better understand the diagnosis made by my doctor | 39 (12.8) | 14 (11.4) | 25 (13.8) | .53 (*χ^2^*) |
|  | Other | 39 (12.8) | 16 (13.0) | 23 (12.7) | .94 (*χ^2^*) |
| **When did you use the Isabel Symptom Checker? (n=304), n (%)** | | | | | <.001 (*χ^2^*) |
|  | Before seeing a doctor | 119 (39.1) | 58 (47.2) | 61 (33.7) | — |
|  | After seeing a doctor | 25 (8.2) | 13 (10.6) | 12 (6.6) | — |
|  | Both before and after seeing a doctor | 113 (37.2) | 27 (22.0) | 86 (47.5) | — |
|  | Instead of seeing a doctor | 47 (15.5) | 25 (20.3) | 22 (12.2) | — |
| **Before I used the Isabel Symptom Checker, I was already planning on going to the doctor (n=304), n (%)** | | | | | .06 (*χ^2^*) |
|  | Strongly disagree | 17 (5.6) | 8 (6.5) | 9 (5.0) | — |
|  | Disagree | 41 (13.5) | 23 (18.7) | 18 (9.9) | — |
|  | Neither agree nor disagree | 100 (32.9) | 43 (35.0) | 57 (31.5) | — |
|  | Agree | 100 (32.9) | 37 (30.1) | 63 (34.8) | — |
|  | Strongly agree | 46 (15.1) | 12 (9.8) | 34 (18.8) | — |
| **Personal financial issues prevented me from seeing a doctor in person for my health issues (n=303), n (%)** | | | | | .09 (*χ^2^*) |
|  | Strongly disagree | 139 (45.9) | 57 (46.7) | 82 (45.3) | — |
|  | Disagree | 97 (32.0) | 43 (35.2) | 54 (29.8) | — |
|  | Neither agree nor disagree | 34 (11.2) | 16 (13.1) | 18 (9.9) | — |
|  | Agree | 25 (8.3) | 5 (4.1) | 20 (11.0) | — |
|  | Strongly agree | 8 (2.6) | 1 (0.8) | 7 (3.9) | — |
| **Policies related to my health insurance prevented me from seeing a doctor in person for my health issues (n=304), n (%)** | | | | | .10 (*χ^2^*) |
|  | Strongly disagree | 142 (46.7) | 55 (44.7) | 87 (48.1) | — |
|  | Disagree | 99 (32.6) | 46 (37.4) | 53 (29.3) | — |
|  | Neither agree nor disagree | 43 (14.1) | 19 (15.4) | 24 (13.3) | — |
|  | Agree | 15 (4.9) | 3 (2.4) | 12 (6.6) | — |
|  | Strongly agree | 5 (1.6) | 0 (0.0) | 5 (2.8) | — |
| **I think the Isabel Symptom Checker gave me useful information for my health problem (n=304), n (%)** | | | | | .53 (*χ^2^*) |
|  | Strongly disagree | 1 (0.3) | 0 (0.0) | 1 (0.6) | — |
|  | Disagree | 6 (2.0) | 1 (0.8) | 5 (2.8) | — |
|  | Neither agree nor disagree | 23 (7.6) | 10 (8.1) | 13 (7.2) | — |
|  | Agree | 184 (60.5) | 79 (64.2) | 105 (58.0) | — |
|  | Strongly agree | 90 (29.6) | 33 (26.8) | 57 (31.5) | — |
| **I experienced positive effects on my health as a result of what I learned from using the Isabel Symptom Checker (n=302), n (%)** | | | | | .03 (*χ^2^*) |
|  | Strongly disagree | 5 (1.7) | 0 (0.0) | 5 (2.8) | — |
|  | Disagree | 14 (4.6) | 3 (2.5) | 11 (6.1) | — |
|  | Neither agree nor disagree | 129 (42.7) | 62 (51.2) | 67 (37.0) | — |
|  | Agree | 114 (37.7) | 44 (36.4) | 70 (38.7) | — |
|  | Strongly agree | 40 (13.2) | 12 (9.9) | 28 (15.5) | — |
| **I experienced positive effects on my finances as a result of what I learned from using the Isabel Symptom Checker (n=303), n (%)** | | | | | .84 (*χ^2^*) |
|  | Strongly disagree | 27 (8.9) | 9 (7.4) | 18 (9.9) | — |
|  | Disagree | 36 (11.9) | 15 (12.3) | 21 (11.6) | — |
|  | Neither agree nor disagree | 172 (56.8) | 69 (56.6) | 103 (56.9) | — |
|  | Agree | 48 (15.8) | 22 (18.0) | 26 (14.4) | — |
|  | Strongly agree | 20 (6.6) | 7 (5.7) | 13 (7.2) | — |
| **When given a list of possible diagnoses for my health problem, I read the relevant information provided by the Isabel Symptom Checker about those diagnoses (n=304), n (%)** | | | | | .46. (*χ^2^*) |
|  | No | 14 (4.6) | 7 (5.7) | 7 (3.9) | — |
|  | Yes | 290 (95.4) | 116 (94.3) | 174 (96.1) | — |
| **After seeing the list of possible diagnoses for my health problem, I used the “Where Now” feature, to help me decide whether to see my doctor or go to the ER^c^ (n=304), n (%)** | | | | | .15 (*χ^2^*) |
|  | No | 203 (66.8) | 88 (71.5) | 115 (63.5) | — |
|  | Yes | 101 (33.2) | 35 (28.5) | 66 (36.5) | — |
| **If the Isabel Symptom Checker advised you to go to the ER, did you follow that advice? (n=304), n (%)** | | | | | .48 (*χ^2^*) |
|  | Not applicable—Isabel Symptom Checker did not suggest I go to the ER | 278 (91.4) | 115 (93.5) | 163 (90.1) | — |
|  | Yes, Isabel Symptom Checker suggested I go to the ER and I went | 14 (4.6) | 5 (4.1) | 9 (5.0) | — |
|  | No, Isabel Symptom Checker suggested I go to the ER, but I decided I didn’t need to go to the ER, but I did go to another medical care provider | 9 (3.0) | 3 (2.4) | 6 (3.3) | — |
|  | No, Isabel Symptom Checker suggested I go to the ER, but I did not go to any medical care provider | 3 (1.0) | 0 (0.0) | 3 (1.7) | — |
| Please rate your overall health before using the Isabel Symptom Checker on a scale of 1-10, with 1 indicating “not in good health” and 10 indicating “in very good health”. (mean, SD) | | 6.2 (2.2) | 7.0 (1.9) | 5.6 (2.2) | — |
| Please rate your overall health after using the Isabel Symptom Checker on a scale of 1-10, with 1 indicating “not in good health” and 10 indicating “in very good health”. (mean, SD) | | 6.7 (2.1) | 7.5 (1.7) | 6.1 (2.1) | — |
| **Some of the diagnoses the Isabel Symptom Checker suggested seemed more alarming than I thought they should have been given my symptoms (n=304), n (%)** | | | | | .002 (*χ^2^*) |
|  | Strongly disagree | 18 (5.9) | 3 (2.4) | 15 (8.3) | — |
|  | Disagree | 88 (28.9) | 24 (19.5) | 64 (35.4) | — |
|  | Neither agree nor disagree | 118 (38.8) | 61 (49.6) | 57 (31.5) | — |
|  | Agree | 71 (23.4) | 31 (25.2) | 40 (22.1) | — |
|  | Strongly agree | 9 (3.0) | 4 (3.3) | 5 (2.8) |  |
| **Some of the diagnoses the Isabel Symptom Checker suggested seemed less alarming than I thought they should have been given my symptoms (n=303), n (%)** | | | | | .04 (*χ^2^*) |
|  | Strongly disagree | 13 (4.3) | 2 (1.6) | 11 (6.1) | — |
|  | Disagree | 88 (29.0) | 28 (23.0) | 60 (33.1) | — |
|  | Neither agree nor disagree | 147 (48.5) | 71 (58.2) | 76 (42.0) | — |
|  | Agree | 50 (16.5) | 19 (15.6) | 31 (17.1) | — |
|  | Strongly agree | 5 (1.7) | 2 (1.6) | 3 (1.7) | — |
| **I am satisfied with the Isabel Symptom Checker as an online diagnostic tool (n=304), n (%)** | | | | | .26 (*χ^2^*) |
|  | Strongly disagree | 2 (0.7) | 0 (0.0) | 2 (1.1) | — |
|  | Disagree | 14 (4.6) | 3 (2.4) | 11 (6.1) | — |
|  | Neither agree nor disagree | 25 (8.2) | 13 (10.6) | 12 (6.6) | — |
|  | Agree | 151 (49.7) | 64 (52.0) | 87 (48.1) | — |
|  | Strongly agree | 112 (36.8) | 43 (35.0) | 69 (38.1) | — |
| **I think the Isabel Symptom Checker was easy to use (n=303), n (%)** | | | | | .06 (*χ^2^*) |
|  | Strongly disagree | 0 (0.0) | 0 (0.0) | 0 (0.0) | — |
|  | Disagree | 4 (1.3) | 0 (0.0) | 4 (2.2) | — |
|  | Neither agree nor disagree | 17 (5.6) | 11 (9.0) | 6 (3.3) | — |
|  | Agree | 155 (51.2) | 64 (52.5) | 91 (50.3) | — |
|  | Strongly agree | 127 (41.9) | 47 (38.5) | 80 (44.2) | — |
| **I found the Isabel Symptom Checker very useful as a diagnostic tool (n=301), n (%)** | | | | | .11 (*χ^2^*) |
|  | Strongly disagree | 1 (0.3) | 0 (0.0) | 1 (0.6) | — |
|  | Disagree | 10 (3.3) | 1 (0.8) | 9 (5.0) | — |
|  | Neither agree nor disagree | 37 (12.3) | 20 (16.4) | 17 (9.5) | — |
|  | Agree | 142 (47.2) | 58 (47.5) | 84 (46.9) | — |
|  | Strongly agree | 111 (36.9) | 43 (35.2) | 68 (38.0) | — |
| **The Isabel Symptom Checker provided me with insights that led me closer to a correct diagnosis (n=303), n (%)** | | | | | .01 (*χ^2^*) |
|  | Strongly disagree | 4 (1.3) | 0 (0.0) | 4 (2.2) | — |
|  | Disagree | 16 (5.3) | 3 (2.4) | 13 (7.2) | — |
|  | Neither agree nor disagree | 52 (17.2) | 30 (24.4) | 22 (12.2) | — |
|  | Agree | 150 (49.5) | 59 (48.0) | 91 (50.6) | — |
|  | Strongly agree | 81 (26.7) | 31 (25.2) | 50 (27.8) | — |
| **If I have a medical problem in the future, I would use the Isabel Symptom Checker again (n=304), n (%)** | | | | | .31 (*χ^2^*) |
|  | Strongly disagree | 2 (0.7) | 1 (0.8) | 1 (0.6) | — |
|  | Disagree | 4 (1.3) | 0 (0.0) | 4 (2.2) | — |
|  | Neither agree nor disagree | 20 (6.6) | 11 (8.9) | 9 (5.0) | — |
|  | Agree | 133 (43.8) | 55 (44.7) | 78 (43.1) | — |
|  | Strongly agree | 145 (47.7) | 56 (45.5) | 89 (49.2) | — |
| **I would recommend the Isabel Symptom Checker to a friend or family member who wanted to research likely causes for their symptoms (n=302), n (%)** | | | | | .07 (*χ^2^*) |
|  | Strongly disagree | 2 (0.7) | 0 (0.0) | 2 (1.1) | — |
|  | Disagree | 6 (2.0) | 1 (0.8) | 5 (2.8) | — |
|  | Neither agree nor disagree | 27 (8.9) | 15 (12.4) | 12 (6.6) | — |
|  | Agree | 139 (46.0) | 62 (51.2) | 77 (42.5) | — |
|  | Strongly agree | 128 (42.4) | 43 (35.5) | 85 (47.0) | — |
| **If asked, my doctor would support my decision to use the Isabel Symptom Checker (n=300), n (%)** | | | | | .02 (*χ^2^*) |
|  | Strongly disagree | 9 (3.0) | 0 (0.0) | 9 (5.0) | — |
|  | Disagree | 24 (8.0) | 5 (4.1) | 19 (10.6) | — |
|  | Neither agree nor disagree | 158 (52.7) | 68 (56.2) | 90 (50.3) | — |
|  | Agree | 84 (28.0) | 39 (32.2) | 45 (25.1) | — |
|  | Strongly agree | 25 (8.3) | 9 (7.4) | 16 (8.9) | — |

^a^Not everyone answered every question, so sample sizes vary by variable. In examining variables by the presence or absence of reported diagnostic error, only patients who answered both questions are included.

^b^If a participant used the symptom checker exactly 2 weeks ago, both options would be appropriate, forcing participants to choose one.

^c^ER: emergency room.
