# Supplementary material for: Patient Perspectives on the Usefulness of an Artificial Intelligence–Assisted Symptom Checker: Cross-Sectional Survey Study
Source: J Med Internet Res. 2020 Jan 30;22(1):e14679. doi: 10.2196/14679 (PMC7055765; doi:10.2196/14679)
Supplement: Multimedia Appendix 4 [file jmir_v22i1e14679_app4.docx]

**Multimedia Appendix 4 for Patient Perspectives on Usefulness of an Artificial-Intelligence Assisted Symptom Checker: Cross Sectional Survey Study**

Ashley N.D. Meyer^1^; Traber D. Giardina^1;^ Christiane Spitzmueller^2^; Umber Shahid, DrPH^1^; Taylor M.T. Scott, BA^1^; & Hardeep Singh^1^

^1^Center for Innovations in Quality, Effectiveness and Safety, Michael E. DeBakey Veterans Affairs Medical Center and Baylor College of Medicine, Houston, TX

^2^Department of Psychology, University of Houston, Houston, TX

**Patients’ experiences of discussing the Isabel Symptom Checker results with their physicians**

| Variable^a^ | | | Values | | No reported diagnostic error | | | Reported diagnostic error | *P* value |
| --- | --- | --- | --- | --- | --- | --- | --- | --- | --- |
| **Occurrence of physician visits and discussion of symptom checker** | | | | | | | | |  |
|  | **After you used the Isabel Symptom Checker, did you see a doctor face to face to address the medical issue you were experiencing when you consulted the Isabel Symptom Checker? (N=304), n (%)** | | | | | | | | <.001 (*χ^2^*) |
|  |  | No | 91 (29.9) | | 55 (44.7) | | | 36 (19.9) | — |
|  |  | Yes | 213 (70.1) | | 68 (55.3) | | | 145 (80.1) | — |
|  | **Did you discuss your results from the Isabel Symptom Checker with your doctor? (n=213), n (%)** | | | | | | | | .74 (*χ^2^*) |
|  |  | No | 110 (51.6) | | 34 (50) | | | 76 (52.4) | — |
|  |  | Yes | 103 (48.4) | | 34 (50) | | | 69 (47.6) | — |
| **Why patients chose not to discuss symptom checker results with their doctors** | | | | | | | | | |
|  | **I did not think my doctor would approve of my decision to use the Isabel Symptom Checker (n=110), n (%)** | | | | | | | | .08 (*χ^2^*) |
|  |  | Strongly disagree | 6 (5.5) | | 3 (9) | | | 3 (4) | — |
|  |  | Disagree | 23 (20.9) | | 6 (18) | | | 17 (22) | — |
|  |  | Neither agree nor disagree | 41 (37.3) | | 18 (53) | | | 23 (30) | — |
|  |  | Agree | 29 (26.4) | | 6 (18) | | | 23 (30) | — |
|  |  | Strongly agree | 11 (10.0) | | 1 (3) | | | 10 (13) | — |
|  | **I was worried my doctor would feel I did not trust him/her if I brought information about my results from the Isabel Symptom Checker to my appointment (n=110), n (%)** | | | | | | | | .12 (*χ^2^*) |
|  |  | Strongly disagree | 5 (4.5) | | 2 (6) | | | 3 (4) | — |
|  |  | Disagree | 31 (28.2) | | 10 (29) | | | 21 (28) | — |
|  |  | Neither agree nor disagree | 34 (30.9) | | 15 (44) | | | 19 (25) | — |
|  |  | Agree | 27 (24.5) | | 6 (18) | | | 21 (28) | — |
|  |  | Strongly agree | 13 (11.8) | | 1 (3) | | | 12 (16) | — |
|  | **I did not want my doctor to feel I was trying to second guess or replace him/her by using the Isabel Symptom Checker (n=108), n (%)** | | | | | | | | .57 (*χ^2^*) |
|  |  | Strongly disagree | 4 (3.7) | | 2 (6) | | | 2 (3) | — |
|  |  | Disagree | 24 (22.2) | | 9 (28) | | | 15 (20) | — |
|  |  | Neither agree nor disagree | 25 (23.1) | | 8 (25) | | | 17 (22) | — |
|  |  | Agree | 38 (35.2) | | 10 (31) | | | 28 (37) | — |
|  |  | Strongly agree | 17 (15.7) | | 3 (9) | | | 14 (18) | — |
| **Experiences of patients who chose to discuss symptom checker results with their doctors** | | | | | | | | | |
|  | **My doctor was interested in learning about my results from the Isabel Symptom Checker (n=103), n (%)** | | | | | | | | .04 (*χ^2^*) |
|  |  | Strongly disagree | 8 (7.8) | | 1 (3) | | | 7 (10) | — |
|  |  | Disagree | 16 (15.5) | | 2 (6) | | | 14 (20) | — |
|  |  | Neither agree nor disagree | 37 (35.9) | | 11 (32) | | | 26 (38) | — |
|  |  | Agree | 36 (35.0) | | 16 (47) | | | 20 (29) | — |
|  |  | Strongly agree | 6 (5.8) | | 4 (12) | | | 2 (3) | — |
|  | **My doctor was dismissive of the Isabel Symptom Checker and similar tools (n=103), n (%)** | | | | | | | | .20 (*χ^2^*) |
|  |  | Strongly disagree | 12 (11.7) | | 6 (18) | | | 6 (9) | — |
|  |  | Disagree | 38 (36.9) | | 13 (38) | | | 25 (36) | — |
|  |  | Neither agree nor disagree | 36 (35.0) | | 13 (38) | | | 23 (33) | — |
|  |  | Agree | 10 (9.7) | | 2 (6) | | | 8 (12) | — |
|  |  | Strongly agree | 7 (6.8) | | 0 (0) | | | 7 (10) | — |
|  | **My use of the Isabel Symptom Checker provided my doctor with relevant and helpful information (n=103), n (%)** | | | | | | | | .53 (*χ^2^*) |
|  |  | Strongly disagree | 3 (2.9) | | 0 (0) | | | 3 (4) | — |
|  |  | Disagree | 7 (6.8) | | 2 (6) | | | 5 (7) | — |
|  |  | Neither agree nor disagree | 43 (41.7) | | 12 (35) | | | 31 (45) | — |
|  |  | Agree | 42 (40.8) | | 17 (50) | | | 25 (36) | — |
|  |  | Strongly agree | 8 (7.8) | | 3 (9) | | | 5 (7) | — |
|  | **My doctor seemed open to discussing my results from the Isabel Symptom Checker (n=103), n (%)** | | | | | | | | .10 (*χ^2^*) |
|  |  | Strongly disagree | 6 (5.8) | | 0 (0) | | | 6 (9) | — |
|  |  | Disagree | 15 (14.6) | | 6 (18) | | | 9 (13) | — |
|  |  | Neither agree nor disagree | 20 (19.4) | | 3 (9) | | | 17 (25) | — |
|  |  | Agree | 50 (48.5) | | 20 (59) | | | 30 (43) | — |
|  |  | Strongly agree | 12 (11.7) | | 5 (15) | | | 7 (10) | — |
|  | **Based on my interactions with my doctor, I feel encouraged to use the Isabel Symptom Checker again in the future (n=103), n (%)** | | | | | | | | .74 (*χ^2^*) |
|  |  | Strongly disagree | 2 (1.9) | | 0 (0) | | | 2 (3) | — |
|  |  | Disagree | 5 (4.9) | | 2 (6) | | | 3 (4) | — |
|  |  | Neither agree nor disagree | 26 (25.2) | | 7 (21) | | | 19 (28) | — |
|  |  | Agree | 45 (43.7) | | 17 (50) | | | 28 (41) | — |
|  |  | Strongly agree | 25 (24.3) | | 8 (24) | | | 17 (25) | — |
|  | **Did you bring a printout of your results from the Isabel Symptom Checker to your doctor's appointment? (n=103), n (%)** | | | | | | | | .83 (*χ^2^*) |
|  |  | No | | 86 (83.5) | | 28 (82) | 58 (84) | | — |
|  |  | Yes | | 17 (16.5) | | 6 (18) | 11 (16 | | — |

^a^Not everyone answered every question, so sample sizes vary by variable. In examining variables by the presence or absence of reported diagnostic error, only patients who answered both questions are included.
