# Supplementary material for: Patient Perspectives on the Usefulness of an Artificial Intelligence–Assisted Symptom Checker: Cross-Sectional Survey Study
Source: J Med Internet Res. 2020 Jan 30;22(1):e14679. doi: 10.2196/14679 (PMC7055765; doi:10.2196/14679)
Supplement: Multimedia Appendix 5 [file jmir_v22i1e14679_app5.docx]

**Multimedia Appendix 5 for Patient Perspectives on Usefulness of an Artificial-Intelligence Assisted Symptom Checker: Cross Sectional Survey Study**

Ashley N.D. Meyer^1^; Traber D. Giardina^1;^ Christiane Spitzmueller^2^; Umber Shahid, DrPH^1^; Taylor M.T. Scott, BA^1^; & Hardeep Singh^1^

^1^Center for Innovations in Quality, Effectiveness and Safety, Michael E. DeBakey Veterans Affairs Medical Center and Baylor College of Medicine, Houston, TX

^2^Department of Psychology, University of Houston, Houston, TX

**Additional Findings from Open-Ended Responses**

**Please describe any positive or negative health consequences you experienced based on what you learned from using the Isabel Symptom Checker** (n=175). Summarizing the general valence of responses, the majority were positive (96%; 168/175), leaving few negative responses (4%; 7/175). The most-often talked about health consequence of using the symptom-checker was that people were able to determine whether their condition might be serious, helping them distinguish when to seek medical attention based on their symptoms and severity (28%; 49/175). The next most-often talked about consequence people reported was gaining information or knowledge about possible diagnoses or symptoms (19%; 34/175). The next most frequent type of response included the idea that the symptom-checker led people to follow-up with a healthcare professional or on test results (18%; 32/175). These consequences were followed in prevalence by experiences of relief, including feelings of reassurance or relaxation (15%; 26/175); condition management, including learning how to alleviate symptoms or provide at-home remedies (15%; 26/175), attainment of knowledge about a condition they were already diagnosed with (14%; 24/175), learning about who they should talk to (e.g., a specialist or a different doctor; 6%; 11/175), and acquiring a second opinion (2%; 4/175).

**Please describe any positive or negative financial consequences you experienced based on what you learned from using Isabel Symptom Checker** (n=69)**.** Summarizing the general valence of responses, the majority were positive (93%; 64/69), leaving few negative responses (7%; 3/69). The most-often talked about financial consequence of using the symptom-checker was that people reported making fewer doctor visits after using the symptom-checker than they would have otherwise (49%; 34/69). This was followed in prevalence by the financial consequence of saving money, for example through the prevention of additional diagnostic tests (14%; 10/69). Additional consequences included being able to determine which type of physician to see (saving money from unnecessary referrals; 13%; 9/69), helping them determine which expenses were necessary for treatment (6%; 4/69), increasing medical costs (4%; 3/69), and an inability to afford going to the doctor (1%; 1/69).

**Please describe your interaction with your doctor when discussing your Isabel Symptom Checker results below** (n=29)**.** Slightly more than half of responses were positive (52%; 15/29): slightly less than half were negative (48%; 14/29). The most-often talked about type of experience discussing symptom-checker results with physicians was frustration on the part of the patients during the discussion (24%; 7/29), followed by the perception that physicians were open to the use of Isabel (21%; 6/29). This was followed by the experience physicians were dismissive (17%; 5/29), followed by the experience that findings from the symptom-checker helped guide patients in their conversations with their physicians (10%; 3/29). Additionally, people reported feeling empowered having the results to discuss with their physicians, which allowed them to advocate for themselves (7%; 2/29) and they reported that the discussion made physicians take action (7%; 2/29).

**Please elaborate on why you chose not to discuss your use of Isabel Symptom Checker with your doctor below** (n=52)**.** For those who reported going to the doctor after using Isabel, but who chose not to discuss the Isabel findings with their physicians, a large portion of the respondents reported being worried about pushback or having concerns about their physicians’ reactions (40%; 21/52). Another large group of respondents commented that they were merely using the tool to inform themselves about what to ask the doctor or how to handle the condition and not to discuss it directly (21%; 11/52). To a lesser extent, people conveyed that they chose not to discuss the results because they thought it was unnecessary (e.g., because the physician and Isabel came to the same conclusion; 12%; 6/52), because they wanted confirmation that the doctor would come up with the same diagnosis or come up with an unbiased diagnosis (12%; 6/52), because they thought the symptom-checker results were too general or unhelpful to discuss (10%; 5/52), or because they were concerned about the little bit of time they already had with their physicians and did not feel they had time to discuss the results (6%; 3/52).

**If you have been misdiagnosed and would like to share your story, please do so in the following textbox. This might help us figure out ways to help patients like you in the future. All of the information will be kept confidential** (n=108)**.**

The sample of patients who shared diagnostic error stories consisted mostly of females (82.4%; 89/108) and Caucasians/whites (91.7%; 99/108), with a mean age of 48.8 years (SD=16.6). Patients reported several types of clinical conditions where they experienced diagnostic errors: rheumatological/immunological (32.4%; 35/108; most commonly Fibromyalgia), infectious (10.2%; 11/108), gastroenterological (7.4%; 8/108), reproductive (7.4%; 8/108), cancerous (6.5%; 7/108), endocrine (4.6%; 5/108; most commonly hypothyroidism), cardiovascular (2.8%; 3/108), respiratory (2.8%; 3/108), and mental health-related conditions (2.8%; 3/108). An additional 11.1% (12/108) of patients in this group reported being incorrectly labelled with mental health issues when they became frustrated, anxious, or depressed after prolonged waits to obtain correct diagnoses and multiple referrals.

Patients perceived several contributory factors as causing their diagnostic errors including 1) physicians’ inability to manage diagnostic uncertainty (30.6%; 33/108), 2) physicians making multiple unnecessary referrals to others when faced with challenging diagnoses (20.4%; 22/108) rather than thoughtfully connecting the dots between diagnostic data themselves, 3) physicians prioritizing financial gains versus patient benefit (17.6%; 19/108), 4) physicians unfairly labelling patients (e.g., as drug or attention seekers, “drama-queens”, as having symptoms ‘all in [their] head[s]’, or as not ‘look[ing] sick’: 14.8%; 16/108), and 5) physicians not taking time to listen to patients (12.0%; 13/108). Several patients reported harm, including long-term health consequences from errors, such as disability or life-threatening experiences (67.6%; 73/108).

**Additional Behavioral Differences as Related to Demographics**

Likelihood of going to the ER when the symptom-checker suggested was not significantly related to gender (53% of females [10/19] and 57% of males [4/7] told to go to the ER did; *P*=~1), income (43% of those with an annual household income <$74,999 [6/14] and 70% of those with higher incomes [7/10] told to go to the ER did; *P*=.24), education (50% of people with less education than a completed college degree [3/6] and 58% of people with at least a college degree [11/19] told to go to the ER did; *P*=~1), or being an underrepresented minority (67% of underrepresented minorities, including American Indian or Alaskan Natives, Blacks or African Americans, and Hispanics or Latinos [2/3], and 56% of others [14/25] told to go to the ER did; *P*=~1).

Similarly, for patients who saw a doctor after using the symptom-checker, likelihood of discussing the results with their doctors was not significantly related to gender (45.0% of females [76/169] and 61% of males [27/44] discussed the results with their doctor; *P*=.06), income (52.1% of those with an annual household income <$74,999 [61/117] and 43% of those with higher incomes [35/81] discussed the results with their doctor; *P*=.25), education (54% of people with less education than a completed college degree [43/80] and 45.0% of people with at least a college degree [59/131] discussed the results with their doctor; *P*=.26), or being an underrepresented minority (73% of underrepresented minorities [11/15] and 46.5% [94/202] of others discussed the results with their doctor; *P*=.06).
